# Evaluation of PCV2 vaccine immunogenicity and efficacy using ELISpot to detect virus-specific memory B cells

**DOI:** 10.1186/s40813-025-00452-7

**Published:** 2025-07-10

**Authors:** Jie Fan, Fangcheng Fan, Zhixiong Chen, Ping Chen, Yanli Zhu, Xin Li, Tiantian Liu, Runcheng Li, Wei Dong, Meng Ge

**Affiliations:** College of Veterinary Medicine, Hunan Agricultural University, Changsha, 410125 China

**Keywords:** Porcine circovirus 2, ELISpot, Memory B cells, Immune memory, Vaccine

## Abstract

**Background:**

Porcine Circovirus 2 (PCV2) vaccination plays a crucial role in preventing porcine circovirus-associated disease (PCVAD). Nevertheless, pig farms face significant challenges in evaluating vaccination efficacy due to the inability of PCV2 vaccines to achieve sterilizing immunity and the variability among vaccine manufacturers. These challenges are further compounded by the limitations of conventional antibody detection methods, which fail to distinguish between maternally-derived antibodies (MDAs) and vaccine-induced antibodies. The accurate evaluation and selection of PCV2 vaccines is critical for the swine industry. The present study aimed to develop an Enzyme-linked immunospot (ELISpot) assay for directly detecting PCV2-specific memory B cells. This approach was used to assess the presence of PCV2-specific memory B cells in piglets with high levels of MDA vaccinated with different PCV2 vaccines, thus enabling the evaluation of vaccine immunogenicity at the cellular level. Furthermore, antibody levels and the viremia status were analyzed using Enzyme-linked immunosorbent assay (ELISA) and quantitative real-time polymerase chain reaction (qPCR) respectively to provide a comprehensive assessment of the ELISpot assay potential for evaluating the vaccine immunogenicity of PCV2 vaccines.

**Results:**

The findings revealed that the optimal conditions for the developed ELISpot assay included stimulation with R848 at a final concentration of 1 µg·mL⁻¹ for three days, a PCV2 Cap protein coating concentration of 1.25 µg·mL⁻¹, a biotinylated goat anti-pig IgG antibody concentration of 5 µg·mL⁻¹, and an HRP-streptavidin concentration of 0.25 µg·mL⁻¹. In high MDA piglets immunized with different vaccines, serum antibody detection showed that PCV2 antibody levels declined continuously over time in all vaccinated and saline-injected control groups, demonstrating similar trends. In contrast, ELISpot analysis demonstrated a significant increase in PCV2-specific memory B cell levels in all three vaccinated groups compared to the saline-injected group. Among the vaccines tested, Vaccine A induced the highest levels of specific memory B cells, followed by Vaccine B. This was consistent with the lower PCV2 infection rates and viremia levels observed in Vaccine A and Vaccine B groups, compared to Vaccine C and saline-injected control groups.

**Conclusions:**

We established an ELISpot assay to quantify PCV2-specific memory B cells, revealing that vaccinated piglets with high MDA levels developed robust memory B cell responses. However, levels of PCV2 IgG antibodies in vaccinated piglets remained statistically indistinguishable from control piglets. These findings demonstrate that ELISpot-based profiling of PCV2-specific memory B cells overcomes the confounding effects of MDA in vaccine efficacy assessments. This approach reliably reflects the humoral immune response induced by vaccination and its relevance in combating natural PCV2 infection, providing valuable guidance for preventing and controlling PCVAD.

## Background

PCV2 was first identified in the 1990s. It is the primary pathogen responsible for post-weaning multisystemic wasting syndrome (PMWS) [1]. It is also associated with various other conditions, including porcine dermatitis and nephropathy syndrome (PDNS), reproductive disorders, enteritis, proliferative and necrotizing pneumonia (PNP), and porcine respiratory disease complex (PRDC). PCV2 is highly prevalent and widely distributed in China and worldwide, causing a substantial impact on swine health and presenting a significant threat to the pig industry [2–4].

PCV2 is the smallest DNA virus known to infect mammals, with a genome length of 1766–1768 bp, encoding 11 Open Reading Frames (ORFs). ORF1 and ORF2 have important biological functions: the former encodes the replication-associated protein (Rep), while the latter encodes the Cap protein, which constitutes the viral capsid. Cap protein is the only structural protein of PCV2, with 60 capsid subunits arranged in 12 pentamers to form an icosahedral viral particle, which is an important target for vaccine development and host immune response studies [5, 6]. Vaccination remains the primary strategy for preventing porcine circovirus-associated disease (PCVAD) [7]. Maintaining a robust immune status in swine herds has been shown to reduce PCV2 prevalence, lower viremia levels and duration, and decrease virus shedding through oral, nasal, and fecal routes [8]. Nonetheless, PCV2 vaccines do not completely protect against infection [9]. In addition, variations in vaccine brands, types, and immunization protocols across farms result in variable herd immunity. Consequently, the timely evaluation of vaccine-induced immunity and the optimization of vaccination programs are essential [10].

Humoral immunity is typically evaluated using ELISA, which measures specific antibody levels. In principle, assessing PCV2-specific antibodies in vaccinated herds can offer valuable insights into vaccine efficacy, particularly in seronegative pigs before vaccination [11–13]. However, the presence of MDA, the complexity of PCVAD, and the unique properties of PCV2 vaccines make antibody-based evaluations complicated. These factors make it difficult to differentiate between MDAs, vaccine-induced antibodies, and antibodies produced in response to virus infections. This challenge limits the accuracy of vaccine efficacy assessments in clinical herds [10–12]. Humoral immune memory can be divided into protective memory, which is mediated by persistent antibodies or plasma cells, and reactive memory, which is mediated by memory B cells. Memory B cells play a pivotal role in immune responses by proliferating and differentiating into plasma cells upon re-exposure to an antigen, producing antibodies and long-lived plasma cells. As the cornerstone of humoral immune memory, memory B cells serve as a reliable indicator of humoral immunity and directly detecting these cells provides an accurate assessment of immune responses [14, 15].

Current approaches to evaluate the immunogenicity of PCV2 vaccines in pigs primarily focus on assessing cell-mediated immunity through T-cell ELISpot assays that detect IFN-γ -secreting cells [16–19]. Nevertheless, there are no reported studies in the literature that evaluate humoral immune responses using PCV2-specific B cell ELISpot assays. ELISpot is a well-established method for detecting memory B cells [20], offering both high specificity and sensitivity [21]. The present study sought to develop an ELISpot assay for detecting PCV2-specific memory B cells. The assay was employed to monitor dynamic changes in specific memory B cells following PCV2 vaccination under conditions of high MDA levels and to evaluate the immune response induced by the vaccine.

## Methods

### Animals

The experimental animals were divided into two batches. The first batch included four clinically healthy 90-day-old pigs, which were PCV2 seronegative and virus-negative (serum qPCR negative) before vaccination. All four pigs in this group were immunized with a single intramuscular injection of Ingelvac CircoFLEX^®^ vaccine (Boehringer Ingelheim, Germany). Blood samples were collected by Anterior vena cava puncture at 14 days post-vaccination (dpv) using heparinised tubes (BD Vacutainer^®^, USA), and Peripheral Blood Mononuclear Cells (PBMCs) were isolated for the development of the PCV2 ELISpot method. The pigs in this group were housed in a single pen equipped with concrete flooring, containing a stainless steel feeder and swine-specific drinking nipples to allow ad libitum access to feed and water. The facility was ventilated with exhaust fans, maintaining an indoor humidity of 70 ± 5% and a constant ambient temperature of 25 °C. Each pig received approximately 1.5 kg of feed daily.

**Fig. 1:**
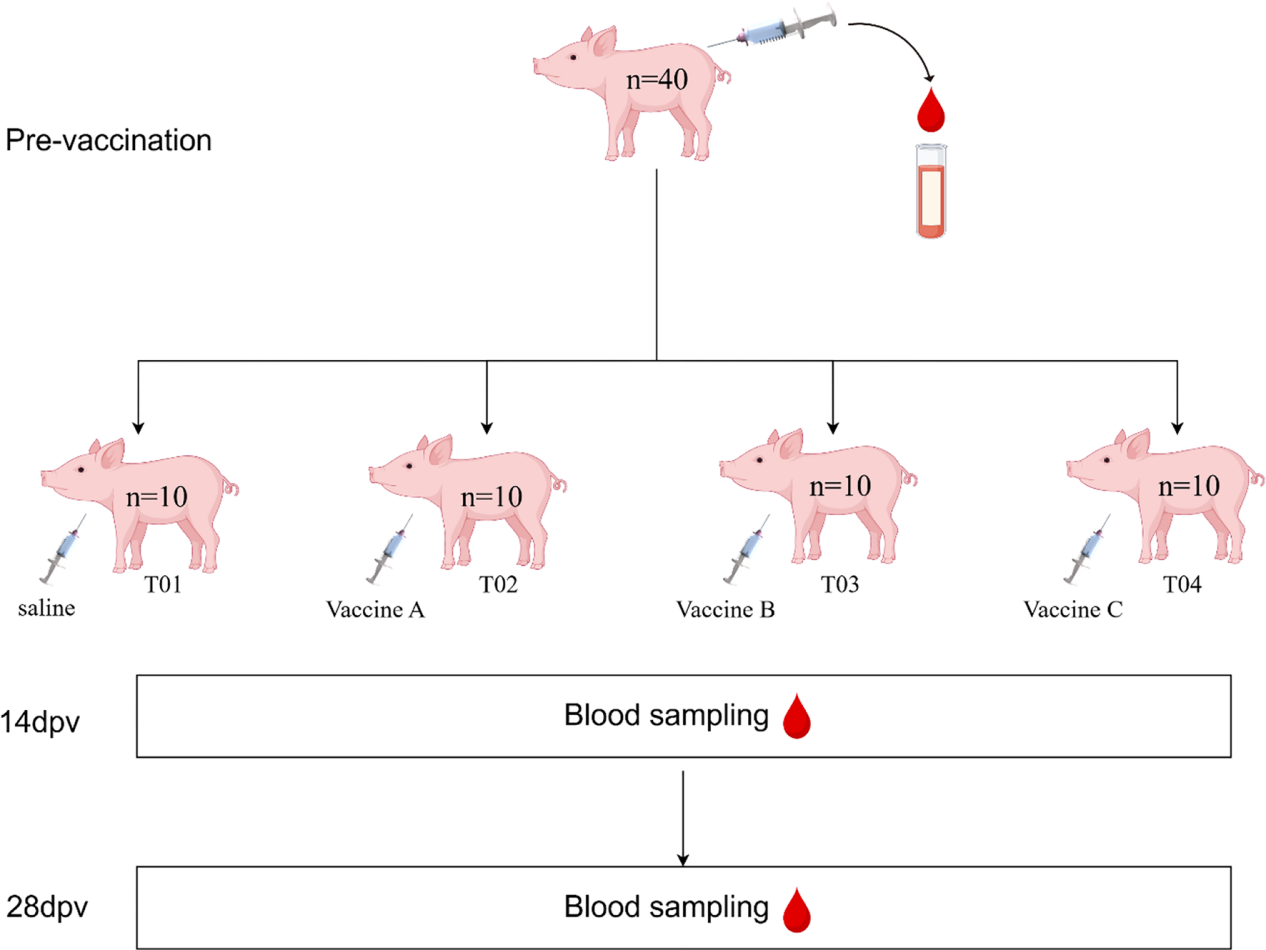
Experimental design of the second batch of pig vaccination including the experimental groups. T01: Saline group; T02: Vaccine A; T03: Vaccine B; T04: Vaccine C. Pre-vaccination, 14 dpv and 28 dpv for blood collection. The experiment ended at 28 dpv (figure prepared by using Figdraw)

The second batch consisted of 40, four-week-old weaned piglets with relatively high maternal PCV2 antibody levels. These piglets were divided into four groups (T01-T04), with 10 piglets in each group. Group T01 was the control group, which injection was a volume of saline equivalent to the vaccine volume injected to vaccinated groups. Group T02 was vaccinated with Vaccine A, a subunit vaccine produced by Company A, which is based on a baculovirus expression system. Group T03 received Vaccine B, a baculovirus-derived subunit vaccine produced by Company B. Group T04 was vaccinated with Vaccine C, an inactivated vaccine produced by Company C. All groups were immunized via intramuscular injection. Blood samples (3–5 mL) were collected by Anterior vena cava puncture using heparinised tubes (BD Vacutainer^®^, USA) before vaccination, 14 days dpv and 28 dpv (Fig. 1). The four experimental groups were housed in separate compartments within the same room of an intensive farming facility, equipped with exhaust fans and cooling pads. Environmental conditions were maintained at 70 ± 5% relative humidity and 25 °C ambient temperature. Pigs were provided with approximately 0.3 kg of feed per animal daily.​.

### Isolation of porcine PBMC

PBMCs were isolated from blood collected in lithium-heparin by the PBMC isolation kit for pigs (Solarbio, China). In brief, cells were washed thrice with PBS containing 3% FBS (Thermo Fisher Scientific, USA), resuspended in RPMI-1640 (Thermo Fisher Scientific, USA) medium supplemented with 10% FBS and 1% Penicillin-Streptomycin, and subjected to Trypan Blue (Solarbio, China) exclusion assay to confirm viability > 98%. After quantifying cell density, cells were diluted to 1 × 10^6^ cells·mL^−^¹ and seeded 2.5 × 10^6^ cells into 6-well polystyrene plates for cell culture (LABSELECT, China). Plates were incubated at 37 °C with 5% CO_2_. Cell samples not processed immediately were stored in liquid nitrogen in 10% DMSO + 90% FBS.

### ​Establishment of the ELISpot assay for PCV2-specific memory B cells

The PBMCs were isolated from pigs vaccinated with Ingelvac CircoFLEX^®^ vaccine, counted using a cell counter (Countstar Mira FL Pro, China), and seeded into 6-well cell culture plates at 2.5 mL per well at a cell count of 1 × 10^6^· mL^− 1^. TLR agonist imidazoquinoline resiquimod (R848; Solarbio, China) was added to each well at a final concentration of 1 µg·mL⁻¹ and gently mixed. The cells were incubated at 37 °C in a 5% CO₂ incubator, with stimulation times set at 1, 2, 3, 4, 5, and 6 days. After each respective stimulation period, 18 µL of stimulated lymphocytes were mixed with 2 µL of 0.4% trypan blue and stained for 3 min (final trypan blue concentration 0.04%) to count viable cells. Concurrently, PBMCs from each stimulation period were subjected to the ELISpot assay to determine the optimal stimulation time. Cap protein, prepared and stored in the laboratory [22], was serially diluted to 100, 50, 25, 12.5 and 6.25 µg·mL⁻¹ in PBS buffer and individually coated onto ELISpot plates (Mabtech, Sweden). Four replicate wells and one control well were included for each condition to determine the optimal coating concentration. Biotinylated goat anti-pig IgG antibody (Solarbio, China) was diluted two-fold serially with PBS containing 0.5% FBS, and the ELISpot assay was performed with four replicate wells and one control well for each condition to identify the optimal concentration of the biotin-labeled secondary antibody. Similarly, HRP-streptavidin (Solarbio, China) was diluted two-fold serially with PBS containing 0.5% FBS, and the ELISpot assay was conducted with four replicate wells and one control well for each condition to determine the optimal concentration of the HRP-labeled streptavidin. ​​Concurrently, ​​PBMCs without R848 stimulation served as negative controls.​ ELISpot results were quantified using AID ELISPOT Reader-iSpot (AID-Autoimmun Diagnostika GmbH, Germany) with the following parameters: Intensity min 20, Size min 80, Invert recognition OFF (dark objects on bright background), and Quality-Speed Count Relation set to high-low.

### ELISA detection of PCV2-specific IgG antibodies

PCV2-specific IgG antibodies in 116 serum samples from the second batch of pigs were detected using an in-house-developed ELISA method [22]. High-binding 96-well microtiter plates (Corning Costar, USA) were coated with Cap protein diluted to 0.5 µg· mL^− 1^ in carbonate coating buffer (pH 9.6). After washing the plates with PBST (PBS containing 0.05% Tween-20), they were blocked with PBST containing 5% dried skim milk at 37 °C for 2 h and washed again with PBST. Collected serum samples and reference sera (antibody-negative and positive control sera were analyzed using the BioChek PCV2 ELISA kit) were diluted at a ratio of 1:100 and 100 µL per well were added and incubated at 37 °C for 30 min. Following incubation, the plates were washed with PBST, and goat anti-pig IgG/HRP antibody (diluted 1:8000, KPL, USA) was added at 100 µL per well, with incubation at 37 °C for 30 min. The plates were washed with PBST before adding 50 µL of 3,3,5,5-tetramethylethylenediamine solution (SureBlue Reserve TMB Microwell Peroxidase Substrate; KPL, USA) substrate per well and incubating them in the dark at 37 °C for 15 min. The reaction was stopped by adding 50 µL of 2 mol· L^− 1^ H₂SO₄ per well. Absorbance was measured at OD_450nm_ using a microplate reader (Thermo Fisher Scientific, USA), and the S/P value (sample OD_450nm_ - negative-control OD_450nm_/positive-control OD_450nm_ - negative-control OD_450nm_) was calculated.

### ELISpot detection of PCV2-specific memory B cells

Anticoagulated whole blood collected from the second batch of pigs was used to isolate PBMCs. Memory B cell detection was conducted using the ELISpot method described above. The specificity of memory B cell responses was determined by quantifying the number of immunospots per 1 × 10⁶ PBMCs.

### Quantitative real-time PCR detection of PCV2 viremia

A total of 116 sera from the second batch of experimental pigs were subjected to nucleic acid extraction using a nucleic acid extraction kit (Bioer, China) at pre-vaccination, 14 dpv and 28 dpv. Primers and probes targeting the PCV2-ORF2 region were specifically designed based on the PCV2 gene sequence available in GenBank (KU317478.1). The sequences were as follows: PCV2-F:5’- AAAGAAGTGCGCTGTAAGTATTACC-3’, PCV2-R: 5’-GCATATTGCTGCTGAGGTGCT-3’, and Probe: FAM- CACTTCGGCAGCGGCAGCAC-BHQ1. Quantitative PCR (qPCR) was performed using real-time PCR instrument (Bioer, FQD-96 A, China) under the following thermal cycling conditions: an initial step at 37 °C for 2 min, followed by 95 °C for 30 s, and then 45 cycles of 95 °C for 10 s and 60 °C for 30 s. Fluorescence signals were recorded during each extension phase to generate amplification curves and determine CT values. The viral copy number of each sample was calculated using the PCV2 standard curve established in the laboratory, and three replicates of each sample were performed.

### Statistical analysis

ELISpot and ELISA experimental data were statistically analyzed using GraphPad Prism 8.0.2. Initially, normality and homogeneity tests were performed to determine whether the data conformed to a normal distribution. Subsequently, one-way analysis of variance (one-way ANOVA) was used to compare data across multiple groups. P-value < 0.05 was considered statistically significant, *P* ≥ 0.05 indicated no significant difference, and *P* < 0.01 denoted a highly significant difference.

For qPCR results, the viral load is calculated based on the CT value of the sample using a previously established standard curve with the equation: X = 10^(38.77− CT / 3.1761)^. The coefficient of determination R² was equal to 0.9943. Each sample was tested in triplicate, and the final result is presented as the mean of the three replicates.

## Results

### Stimulation duration of PBMCs by R848

**Fig. 2:**
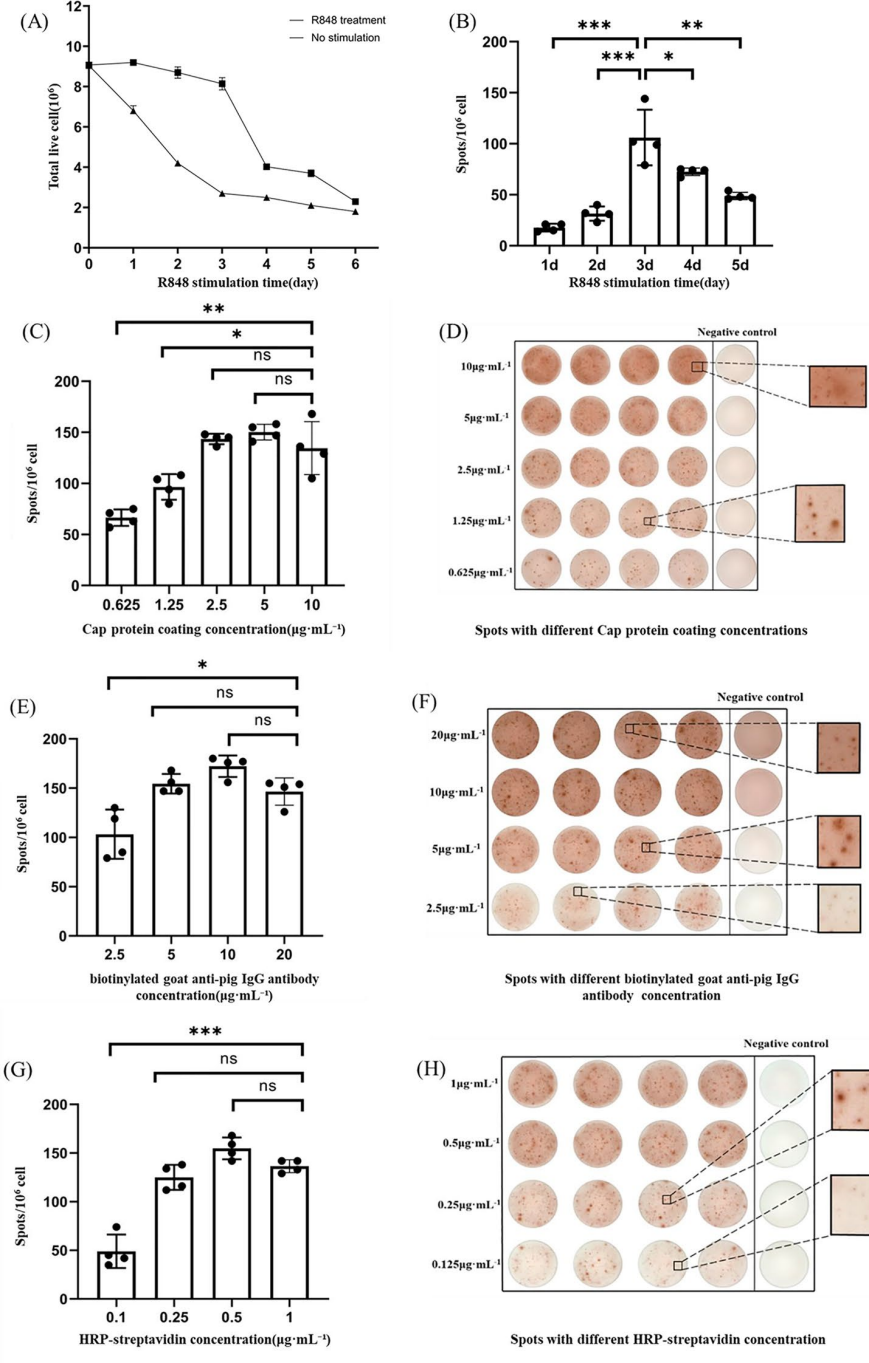
Optimization of ELISpot conditions for detecting PCV2-specific memory B cells. **A**: The influence of R848 stimulation time on PBMCs survival; **B**: The influence of R848 stimulation time on the number of PCV2 antibodies-secreting cells; **C**: Effect of Cap protein coating concentration on spot number; **D**: Effect of Cap protein coating concentration on spot and background colour, with specific spot morphology and background colour shown in black boxes. Negative control (NC) refers to unstimulated PBMCs; **E**: Effect of biotinylated goat anti-pig IgG antibody concentration on spot number; **F**: Effect of biotinylated goat anti-pig IgG concentration on spot and background colour, with specific spot morphology and background colour shown in black boxes, NC refers to unstimulated PBMCs; **G**: Effect of HRP-streptavidin concentration on the number of spots. **H**: Effect of HRP-streptavidin concentration on spot and background colour, with specific spot morphology and background colour shown in black boxes, NC refers to unstimulated PBMCs. ***, *p* < 0.001; **, *p* < 0.01; * *p* < 0.05; ns, *p* ≥ 0.05

Experimental results revealed that stimulation with R848 significantly enhanced the number of viable PBMCs (Fig. 2A). After 3 days of R848 stimulation at a concentration of 1 µg·mL⁻¹, the differentiation of antigen-specific B cells reached its peak, with levels markedly higher than those observed in the other groups (Fig. 2B). Therefore, by taking into account factors such as the number of viable cells and the number of PCV2-specific B cells generated by stimulation, the optimal stimulation conditions for the ELISpot assay were determined to be 3 days of stimulation with R848 at a final concentration of 1 µg·mL⁻¹ when both the number of viable cells and the number of activated memory B cells were at a higher level.

### Coating concentration of PCV2 cap protein

The background color intensity in the ELISpot assay trends with the coating protein concentration. When the coating protein concentration was reduced to 1.25 µg·mL⁻¹, the background color lightened, and the edges of the specific spots appeared clear and well-defined, with no smearing, making them easy to distinguish. Within the concentration range of 2.5 to 10 µg·mL⁻¹, the number of spots did not differ significantly (Fig. 2C). However, at a concentration of 2.5 µg·mL⁻¹, the background was minimal, and the spots were both clear and easy to count (Fig. 2D). Therefore, considering the number of spots and the background colour, the Cap concentration of 1.25 µg·mL^− 1^ was chosen as the optimal concentration of protein.

### The optimal concentration of biotinylated goat anti-pig IgG antibody

For biotinylated goat anti-pig IgG antibody at concentrations ranging from 2.5 to 10 µg·mL⁻¹, the background color intensified as the concentration increased, and the number of spots correspondingly increased. However, when the biotinylated goat anti-pig IgG antibody concentration was increased from 2.5 to 20 µg·mL⁻¹, the number of spots decreased, and the staining background became darker, which hindered accurate spot counting. At a concentration of 5 µg·mL⁻¹, the background color remained relatively light, and the spots formed in the wells were clear and well-defined (Fig. 2E and F). Therefore, 5 µg·mL⁻¹ was chosen as the optimal biotinylated goat anti-pig IgG antibody concentration.

### The most suitable concentration of HRP-streptavidin

When the concentration of HRP-streptavidin was set at 1 µg·mL⁻¹ or 0.5 µg·mL⁻¹, the staining background became excessively dark, and at 1 µg·mL⁻¹, the number of spots decreased. ​​In contrast, at a concentration of 0.25 µg·mL⁻¹, reduced background staining with higher antigen-specific spot counts was observed.​ Nonetheless, when the concentration was further reduced to 0.1 µg·mL⁻¹, although the background color significantly lightened, some spots in the wells indicated decreased quality, with unclear edges and a blurred appearance (Fig. 2G and H). Therefore, 0.25 µg·mL⁻¹ was selected as the optimal HRP-streptavidin concentration based on a comprehensive judgement of spot quality, quantity, and background colour.

### Dynamics of PCV2 IgG antibody levels in weaned piglets post-vaccination

**Fig. 3:**
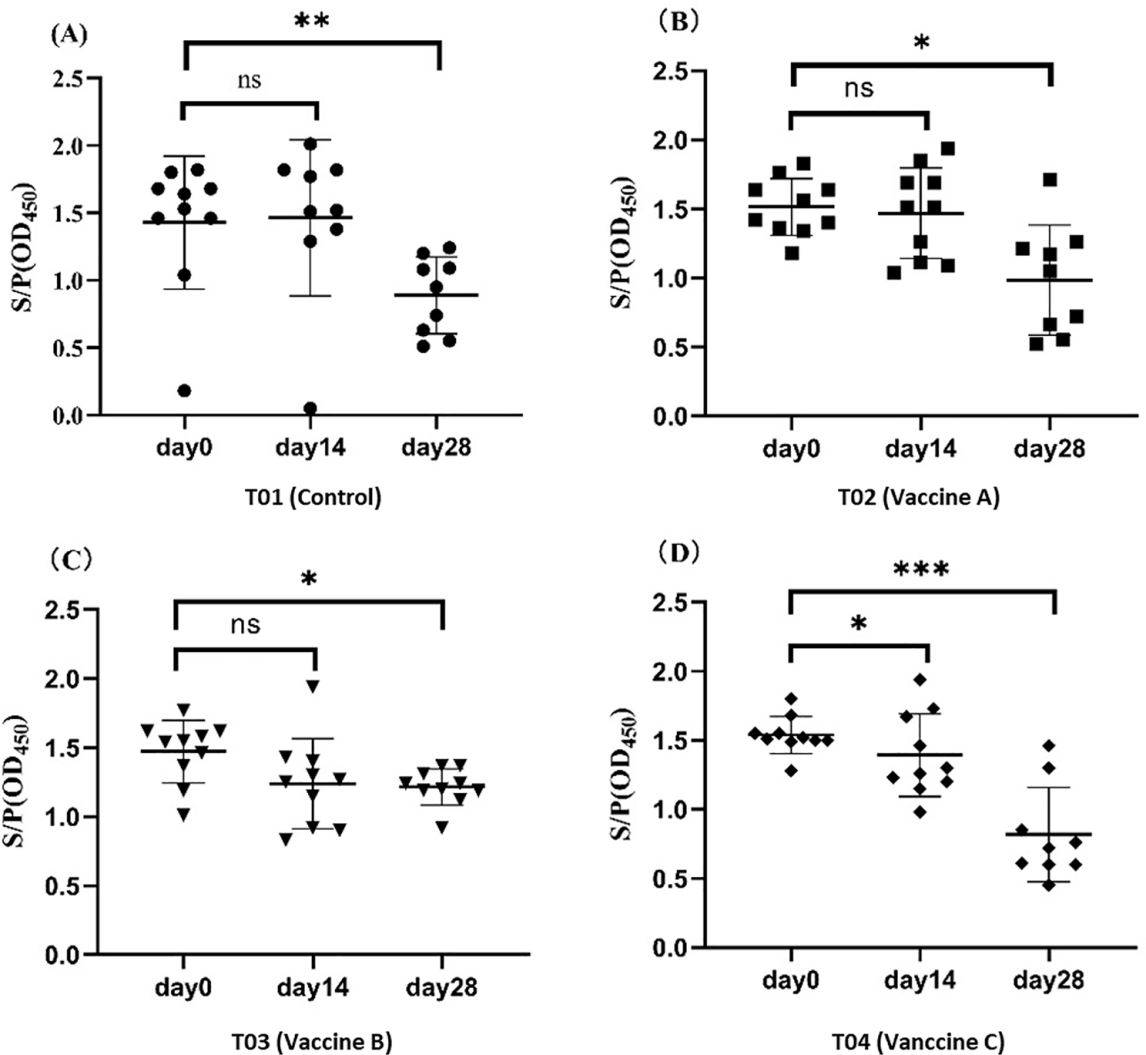
Antibody levels of weaned piglets at various stages after immunization with different PCV2 vaccines. **A**: T01 group (injected with saline); **B**: T02 group (vaccine A); **C**: T03 group (vaccine B); **D**: T04 group (vaccine C). ***, *p* < 0.001; **, *p* < 0.01; * *p* < 0.05; ns, *p* ≥ 0.05

Before vaccination, weaned piglets exhibited high levels of MDA. Fourteen days post-vaccination, antibody levels across all experimental groups showed only slight variations, with no significant differences from pre-vaccination levels. However, by 28 dpv, the antibody levels decreased to varying degrees compared to pre-vaccination levels, and the differences were statistically significant (Fig. 3A, B, C and D).

### Levels of memory B cells in weaned piglets post-vaccination

**Fig. 4:**
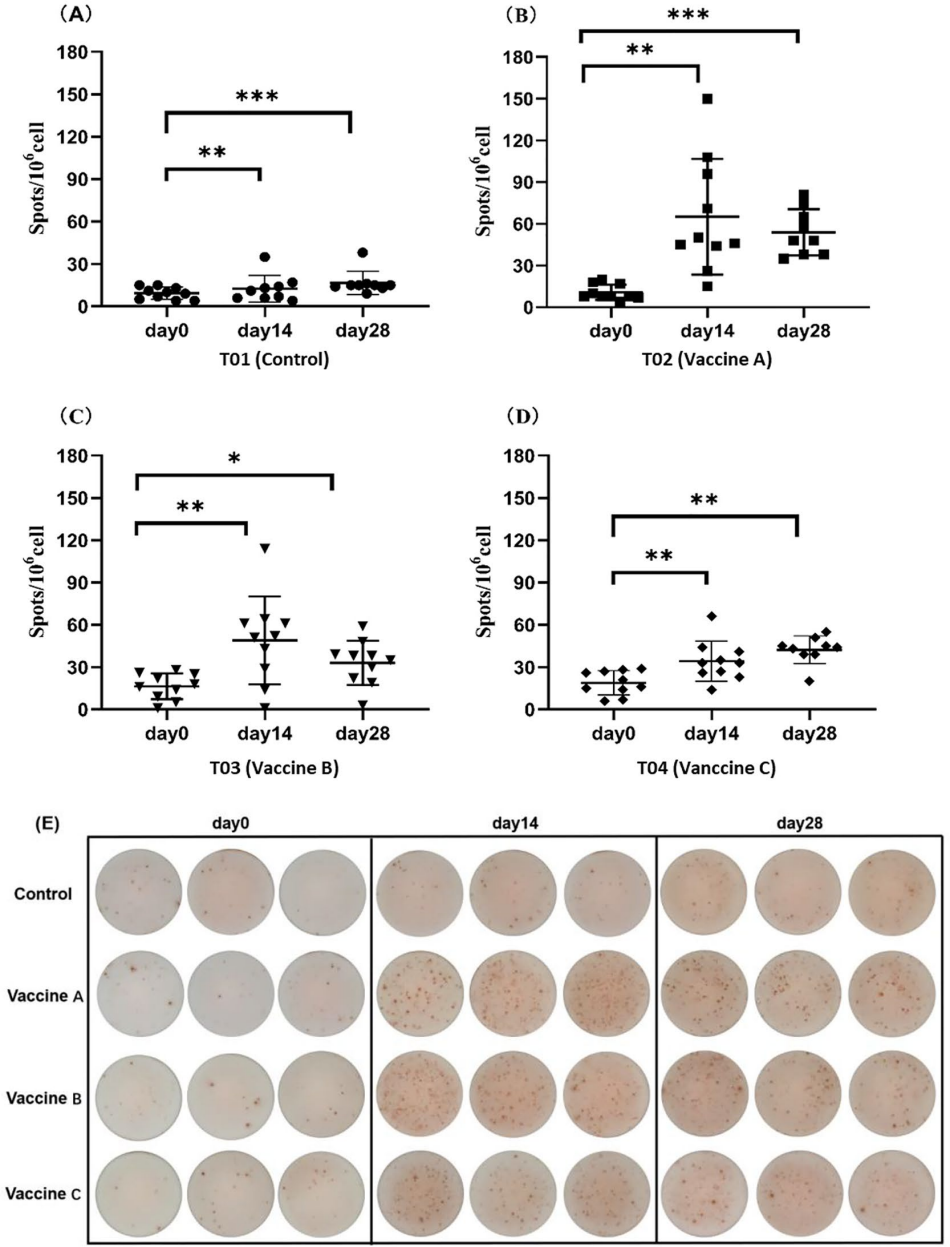
Changes in the number of memory B cells in weaned pigs at various periods after immunization with different PCV2 vaccines. **A**: Group T01 (injected with saline); **B**: Group T02 (vaccine A); **C**: Group T03 (vaccine B); **D**: Group T04 (vaccine C); **E**: ELISpot results of weaned piglets after 0–28 days of immunization with different vaccines. ***, *p* < 0.001; **, *p* < 0.01; * *p* < 0.05; ns, *p* ≥ 0.05

The saline-injected control group (T01) indicated no significant differences in the number of PCV2-specific memory B cells between pre-vaccination, 14 days post-vaccination, and 28 dpv (Fig. 4A). In contrast, groups T02 to T04 demonstrated significant increases in the number of PCV2-specific memory B cells at 14 dpv compared to pre-vaccination levels. Furthermore, the memory B cell counts in T02 to T04 exhibited significantly higher levels compared to the counts recorded in T01 (Fig. 4B, C and D).

### Quantitative real-time PCR identifies PCV2 viremia in weaned piglets post-vaccination

The qPCR results revealed no detectable PCV2 DNA in any serum samples pre-vaccination. At 14 dpv, the T01 group exhibited a positive rate of 11.1%, demonstrating natural PCV2 infection within the herd during this period. By 28 dpv, PCV2 DNA was detectable in serum samples from all groups, indicating varying degrees of PCV2 infection. The infection rates were the lowest in Vaccine A and Vaccine B groups, 11.1% and 10.0%, respectively. In contrast, the T01 and Vaccine C groups had the highest infection rates, both reaching 44.4% (Table 1).

**Table 1:** PCV2 infection rate before and after immunization in different immunization groups and viremia

viremia (PCV2 genomic copies·mL⁻¹)												
Immunization Time	Group	1	2	3	4	5	6	7	8	9	10	Infection Rate
Pre-vaccination	T01	-	-	-	-	-	-	-	-	-	-	0%
	T02	-	-	-	-	-	-	-	-	-	-	0%
	T03	-	-	-	-	-	-	-	-	-	-	0%
	T04	-	-	-	-	-	-	-	-	-	-	0%
14 dpv	T01	-	-	-	-	-	-	6.22 × 10^4^	-	-	Deceased	11.10%
	T02	-	-	-	-	-	-	-	-	-	-	0%
	T03	-	-	-	-	-	-	-	-	-	-	0%
	T04	-	-	-	-	-	-	-	-	-	-	0%
28 dpv	T01	1.24 × 10^0^	2.31 × 10^0^	-	2.49 × 10^0^	-	-	1.43 × 10^3^	-	-	Deceased	44.40%
	T02	Deceased	-	-	3.40 × 10^0^	-	-	-	-	-	-	11.10%
	T03	-	-	-	-	-	-	1.29 × 10^− 1^	-	-	-	10%
	T04	-	1.74 × 10^0^	-	6.92 × 10^− 1^	1.22 × 10^0^	1.58 × 10^0^	-	-	Deceased	-	44.40%

Note: 1–10 represent the numbering of piglets in each group (10 piglets per group)

## Discussion

PCV2 is an environmentally persistent virus with widespread prevalence in global swine populations [23, 24]. Current vaccines fail to induce sterilizing immunity, leaving vaccinated animals susceptible to natural PCV2 infection [25]. In pig production, piglets typically receive their first PCV2 vaccination between 2 and 4 weeks of age, a critical age window when MDAs remain detectable [26]. Understanding the extent to which MDA affects the immunogenicity of PCV2 vaccines, how different vaccines perform in the presence of such interference, and how to predict the protective efficacy of PCV2 vaccines reliably and efficiently are key concerns for pig farms.

To address these knowledge gaps, we developed and optimized an ELISpot assay for detecting PCV2-specific memory B cells. As memory B cells require activation to differentiate into antibody-secreting cells (ASCs), we employed R848 (a TLR7/8 agonist) to stimulate PBMCs. Systematic evaluation revealed that 3-day R848 stimulation optimally balanced ASC differentiation efficiency (peak antibody-secreting cell counts) with cell viability maintenance. Subsequent methodological optimization focused on three key parameters: Cap protein coating concentration (1.25 µg·mL⁻¹ selected), biotinylated detection antibody titration (5 µg·mL⁻¹ optimal), and HRP-streptavidin working concentration (0.25 µg·mL⁻¹ preferred). This optimization significantly improved assay specificity, reducing background staining intensity compared to initial conditions while enhancing spot clarity.

Our longitudinal study tracked PCV2-specific immune responses in MDA-positive weaned piglets using three complementary approaches: ELISpot (memory B cells), ELISA (serum antibodies), and qPCR (viremia detection). ELISA results demonstrated comparable antibody kinetics between vaccinated and non-vaccinated groups (*p* > 0.05 at all timepoints), with both showing progressive antibody decline (Fig. 3A). This aligns with previous reports of MDA-mediated interference, thus obscuring vaccine-induced antibody responses [18, 27], confirming the limited utility of serological assays in MDA-positive populations.

In contrast, ELISpot analysis revealed distinct vaccine-specific differences. All three vaccines induced significant expansion of PCV2-specific memory B cells compared to saline injection, with peak responses at 14 dpv (Fig. 3B). The low number of PCV2-specific memory B cells observed before immunization could be attributed to nonspecific responses or the presence of PCV2-specific lymphocytes transferred via colostrum [28, 29]. Previous research has highlighted significant variations in immune responses induced by vaccines of different types and origins [30, 31]. Herein, subunit vaccines (Vaccine A and Vaccine B) induced higher levels of PCV2 memory B cells after vaccine immunisation compared to inactivated vaccines (Vaccine C), suggesting the superior efficacy of the subunit vaccines in preventing natural PCV2 infection. This conclusion is supported by the qPCR results (Table 1), which revealed lower PCV2 positive rates and viremia levels in pigs vaccinated with subunit vaccines. These findings collectively suggest that the faster and higher the immune memory response induced by the vaccine, the better its protective efficacy, resulting in a lower PCV2 positive rate. Therefore, PCV2-specific memory B cells assessed by ELISpot demonstrated higher accuracy in evaluating vaccine efficacy than ELISA-based detection of PCV2 antibodies, particularly ​​in the presence of MDA. This method offers a solid foundation for making informed vaccine selections on pig farms.

## Conclusions

This study established an optimized ELISpot assay to detect PCV2-specific memory B cells, allowing for precise evaluation of vaccine efficacy even in the presence of significant maternal antibody interference. Furthermore, the study contributed to a deeper understanding of the protective mechanisms of PCV2 vaccines, offering insights that could inform improved strategies for the prevention, control, and potential eradication of PCVAD.

## Data Availability

No datasets were generated or analysed during the current study.
